# Recovery Time, Patient Satisfaction, and Safety of Intranasal Sedatives in Pediatric Dentistry: A Systematic Review and Meta-Analysis

**DOI:** 10.3390/jcm14124038

**Published:** 2025-06-07

**Authors:** Selvakumar Haridoss, Sushmita Shan, Guna Shekhar Madiraju, Kavitha Swaminathan, Rohini Mohan, Faris Yahya I. Asiri, Yousef Majed Almugla, Mohammad Alhussein Hamidaddin

**Affiliations:** 1 Department of Pediatric and Preventive Dentistry, Sri Ramachandra Dental College and Hospital, Sri Ramachandra Institute of Higher Education and Research, Chennai 600116, India; selvakumaarh21@gmail.com (S.H.); d1122001@sriher.edu.in (S.S.); kadva28@gmail.com (K.S.); 2 Department of Preventive Dental Sciences, College of Dentistry, King Faisal University, AlAhsa 31982, Saudi Arabia; yalmugla@kfu.edu.sa (Y.M.A.); mhamidaddin@kfu.edu.sa (M.A.H.); 3 Community Dental Service, Swansea Bay University Health Board, Port Talbot Resource Centre, Port Talbot SA12 7BJ, UK; rohinidr.uk27@gmail.com

**Keywords:** intranasal sedation, midazolam, dexmedetomidine, ketamine, recovery time, patient satisfaction, adverse effects, pediatric patients, systematic review, meta-analysis

## Abstract

**Background:** Intranasal sedation is commonly used in pediatric dentistry to manage dental anxiety and improve patient compliance. This systematic review and meta-analysis aimed to evaluate the recovery time, patient satisfaction, and adverse effects of the intranasal sedatives midazolam, dexmedetomidine, and ketamine in pediatric dental procedures. **Methods**: A systematic search of PubMed, Scopus, the Web of Science, the Cochrane Library, Embase, and Google Scholar was conducted following the PRISMA 2020 guidelines. Only randomized controlled trials (RCTs) involving intranasal sedation in pediatric patients (≤18 years) were included. The revised Cochrane risk of bias tool (RoB 2) was employed to assess study quality. A meta-analysis using a random-effects model was performed to evaluate the recovery time. **Results**: Twenty-one RCTs were included in this review. A meta-analysis of seven studies revealed that dexmedetomidine was associated with significantly longer recovery times compared to midazolam and ketamine. Specifically, midazolam demonstrated the shortest recovery time (mean difference: −19.1 min, *p* < 0.05), followed by ketamine (mean difference: −15.6 min, *p* < 0.05). A qualitative analysis of adverse effects showed mild to moderate complications, including nasal irritation (midazolam), prolonged sedation (dexmedetomidine), and hypersalivation (ketamine). Patient satisfaction was found to be highest with dexmedetomidine, although midazolam was preferred for its faster onset of sedation. **Conclusions**: Intranasal sedation in pediatric dentistry is a safe and effective approach, with each agent exhibiting distinct recovery profiles and safety considerations. The findings emphasize the importance of standardized sedation protocols and the need for further research into the long-term outcomes of these sedatives in pediatric populations.

## 1. Introduction

Pediatric dental anxiety poses a significant challenge in clinical practice, often complicating the management of young patients and limiting the effectiveness of dental treatments [1]. This anxiety among children may arise from factors such as previous negative experiences, the anticipation of pain, unfamiliar environments, and their separation from parents during dental procedures [2]. Effectively managing dental anxiety is crucial as untreated fear can result in the avoidance of dental care, an increased prevalence of oral diseases, and compromised long-term oral health outcomes [3]. Various pharmacological and non-pharmacological approaches have been explored to manage pediatric dental anxiety. While behavior management techniques remain the cornerstone of pediatric dentistry, pharmacological sedation is frequently necessary when conventional methods fail to provide adequate patient cooperation [4].

Among the various sedation options, intranasal sedation has gained increasing popularity due to its non-invasive nature, rapid onset, and efficacy in managing uncooperative pediatric patients [5]. Midazolam and dexmedetomidine are the most commonly used intranasal sedatives, with midazolam acting through the gamma-aminobutyric acid (GABA) system and with dexmedetomidine targeting alpha-2 adrenergic receptors [6,7]. Ketamine is an N-methyl-D-aspartate (NMDA) receptor antagonist with known dissociative anesthetic properties, providing both sedation and analgesia without affecting respiratory function [6]. Moreover, intranasal ketamine is used in children, particularly in specific cases or in combination with other sedatives. In pediatric dentistry, where timely patient cooperation and treatment efficiency are critical, intranasal sedation is especially useful due to its ability to bypass hepatic first-pass metabolism, leading to higher bioavailability and a faster onset of sedation compared to oral or intravenous sedation [8].

Several studies have confirmed the efficacy and safety of intranasal midazolam and dexmedetomidine in pediatric dental settings [9]. A systematic review by Preethy et al. (2021) comparing intranasal midazolam with other sedation routes reported similar sedative efficacy and patient acceptance, with faster onset and recovery compared to oral sedatives [10]. Similarly, dexmedetomidine has been shown to offer a deeper sedative effect with minimal respiratory depression, making it a promising alternative to midazolam in select cases [11]. Despite its advantages, intranasal sedation has limitations. Some studies have reported nasal irritation, transient burning sensations, and unpredictable patient acceptance as potential side effects [12]. Moreover, variability in dosing protocols and inconsistencies in study methodologies have hindered the development of standardized sedation guidelines [13]. Given the growing interest in minimally invasive sedation techniques, further research is needed to consolidate evidence on its safety, recovery times, and patient satisfaction.

While several studies have evaluated the effectiveness of intranasal sedation in pediatric dentistry, previous systematic reviews and meta-analyses have primarily focused on sedation success rates rather than post-sedation recovery parameters [14]. To date, no comprehensive systematic review has exclusively assessed safety, recovery time, and patient satisfaction as primary outcomes. A quantitative synthesis of recovery time is crucial in order to optimize sedation protocols, improve discharge planning, and guide clinical decision-making.

The purpose of this systematic review and meta-analysis was to synthesize existing evidence on the use of intranasal sedation in pediatric dentistry, with a specific focus on evaluating its safety, recovery time, and patient satisfaction. A meta-analysis was conducted solely for recovery time, as the available data on patient satisfaction and adverse effects were insufficient for a statistical synthesis. Through a critical analysis of randomized controlled trials (RCTs) and high-quality studies, this review aimed to provide clinicians with evidence-based insights into the use of intranasal sedation in pediatric dentistry. Additionally, it sought to identify gaps in the current literature and to suggest directions for future research on sedation protocols tailored to pediatric patients.

## 2. Materials and Methods

### 2.1. Study Design and Registration

This systematic review was conducted in accordance with the Preferred Reporting Items for Systematic Reviews and Meta-Analyses (PRISMA) guidelines [15] and the study protocol was registered on PROSPERO (CRD42024601144). Ethical approval was not required for this study, as it involved a systematic review and meta-analysis of existing published literature and data.

### 2.2. Search Strategy

A comprehensive literature search was conducted across six electronic databases: PubMed (Medline), Scopus, the Web of Science, the Cochrane electronic databases, the Excerpta Medica database (EMBASE), and Google Scholar. The search was conducted for relevant studies published between the year 2021 and 28 February 2025, incorporating MeSH terms, free-text keywords, Boolean operators (AND/OR), truncation (*), and phrase searching (“”) to optimize search efficiency. The search was guided by the following PICO question structure: population (P)—pediatric patients (≤18 years) undergoing dental procedures; intervention (I)—intranasal sedation using midazolam, dexmedetomidine, or ketamine; comparison (C)—placebo, oral sedation, or alternative intranasal sedation techniques; and outcome (O)—studies reporting at least one of the following primary outcomes: safety (incidence of adverse effects or complications related to sedation), recovery time (duration, in minutes, required for recovery following sedation), and patient satisfaction (assessed through either qualitative or quantitative measures).

### 2.3. Inclusion and Exclusion Criteria

The inclusion criteria were (1) all randomized controlled trials (RCTs) published in the English language between the year 2021 and 28 February 2025 and (2) a population of between 0 and <18 years. Additional studies were manually searched for by examining the reference lists of all the identified papers. Studies were excluded if they met any of the following criteria: non-randomized studies; case reports; animal studies; studies in which intranasal sedation was used solely as premedication for general anesthesia; investigations employing a combination of sedation techniques (e.g., intranasal plus oral or nitrous oxide); studies with incomplete or missing data regarding the primary outcomes; and studies that did not report at least one of the specified primary outcomes.

### 2.4. Study Selection

The study selection was performed using Rayyan software (https://rayyan.ai/cite), where three authors (SH, SS, and GSM) independently screened and reviewed the titles and abstracts for eligibility. Full-text screening was conducted for the shortlisted studies, and disagreements were resolved by a fourth researcher (KS). Duplicate articles were removed using Rayyan software.

### 2.5. Risk of Bias (RoB 2) Assessment

The revised Cochrane risk of bias tool (RoB 2) was employed to evaluate the quality of the included studies. Two independent reviewers (SS and RM) assessed the bias risk, with any conflicts being resolved by a third reviewer (FYA). Parallel-group RCTs and crossover RCTs were assessed separately using RoB 2. Studies with two or more “Some Concerns” ratings were classified as “High Risk of Bias”. Crossover trials were evaluated for carryover effects, period effects, and outcome measurement bias.

### 2.6. Data Extraction

Two researchers (SH and GSM) independently conducted data extraction using Microsoft Excel. The extracted parameters included the following: authors; the year of publication; sample size; intervention type (midazolam, dexmedetomidine, or ketamine); comparison group; sedation success rates; recovery time; adverse effects; and patient satisfaction. All disagreements were resolved through discussions with a third reviewer (KS). For the purpose of this review, the term “intervention group” refers to the cohort in the included studies who received any of the intranasal sedative agents (midazolam, dexmedetomidine, or ketamine), and the comparator group may have included a placebo, an oral route, or an alternative sedative agent, depending on individual study designs.

### 2.7. Meta-Analysis

A meta-analysis was performed exclusively for the outcome of recovery time, using Review Manager (RevMan 5.4) software. A random-effects model was employed to account for potential heterogeneity among the studies. The effect estimates were reported as mean differences (MD) or standardized mean differences (SMD), depending on the consistency of the measurement scales across the studies. Heterogeneity was assessed using Cochran’s Q-test and the Higgins’ I^2^ statistic to quantify the proportion of total variation due to heterogeneity, the *p*-value associated with the Q-test, and τ^2^ (tau-squared) to estimate the between-study variance within the random-effects model. For three studies, the standard deviations for the outcome of recovery time were calculated based on similar studies as their original values were not reported [16,17,18].

## 3. Results

### 3.1. Study Selection

A comprehensive literature search across six major electronic databases (PubMed, Scopus, the Web of Science, the Cochrane Library, Embase, and Google Scholar) yielded a total of 1431 studies. Following the removal of 311 duplicates, 1120 articles were screened based on their titles and abstracts.

**Figure 1:**
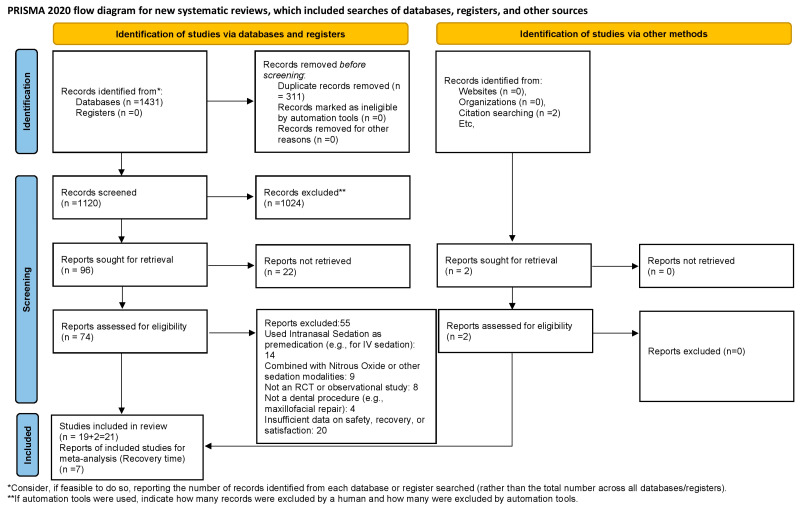
PRISMA flow diagram.

Of these, 1024 studies were excluded for not meeting the predefined inclusion criteria. Subsequently, 96 full-text articles were assessed for eligibility. Following this evaluation, 21 studies met the inclusion criteria and were incorporated into the systematic review. Of these, data from seven studies were suitable for a quantitative synthesis, and a meta-analysis was conducted for the outcome of recovery time. The study selection process is illustrated in the PRISMA flow diagram (Figure 1).

### 3.2. Characteristics of Included Studies

A total of 21 RCTs were included in this review, each investigating the effectiveness of intranasal midazolam, dexmedetomidine, or ketamine for sedation in pediatric dental procedures. Sample sizes varied across studies, ranging from 20 to 118 children, and the dosage regimens differed, reflecting variability in clinical protocols. The studies focused on three primary outcomes: recovery time (analyzed quantitatively), safety (as indicated by the presence of adverse effects), and patient satisfaction (evaluated through qualitative assessments) [16,17,18,19,20,21,22,23,24,25,26,27,28,29,30,31,32,33,34,35,36]. A comprehensive overview of the study characteristics is provided in Table S1 (Supplemental File S1).

### 3.3. Risk of Bias Assessment

**Figure 2:**
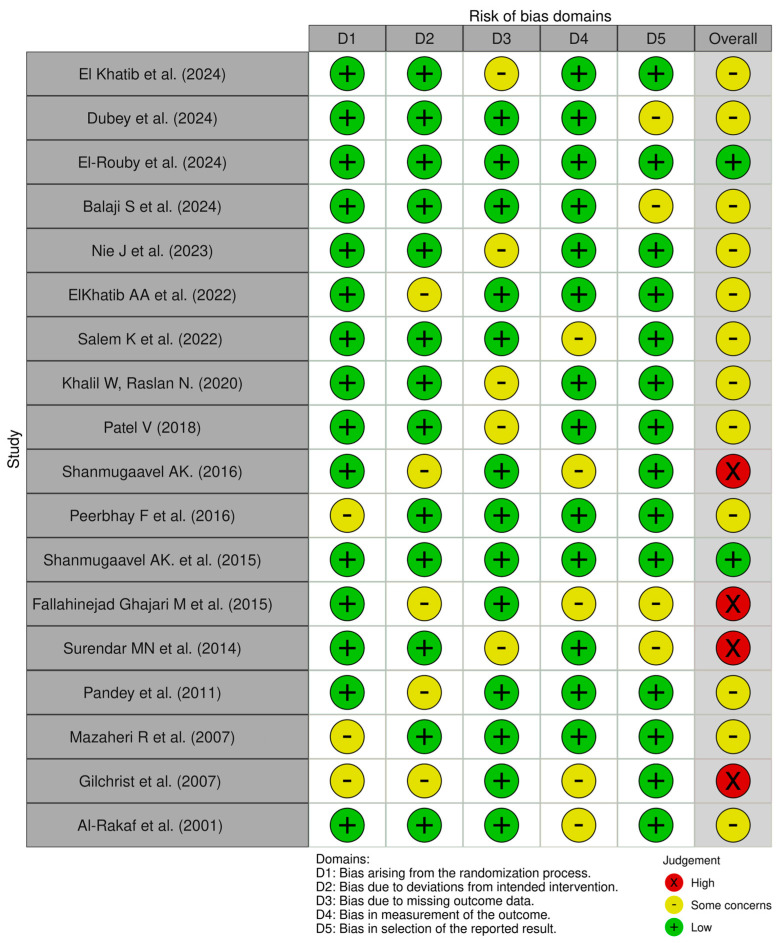
Risk of bias (traffic light plot of included studies: parallel-group RCTs) [16,17,18,19,20,21,22,23,24,25,27,28,29,30,31,33,34,36].

The methodological quality of the included studies was evaluated using Version 2 of the Cochrane risk-of-bias tool for randomized trials [RoB 2] tool. This revealed that two studies were rated as having a low risk of bias [22,31] among the 18 parallel-group RCTs. Twelve studies were judged to have “Some Concerns”, mainly due to issues in the measurement of the outcome and the selection of the reported results [16,19,20,22,23,24,25,26,27,28,33,34,36]. The remaining four studies were classified as having a high risk of bias, primarily due to missing outcome data or deviations from the intended intervention protocols (Table 1) (Figure 2).

**Figure 3:**
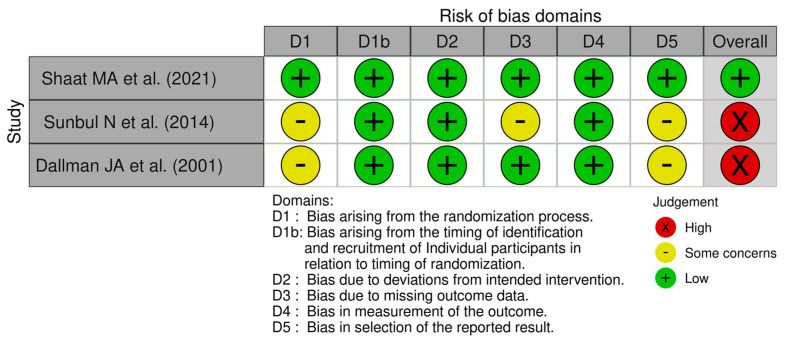
Risk of bias (traffic light plot of included studies-Crossover RCTs) [26,32,35].

In addition, three crossover RCTs were evaluated separately for carryover effects, period effects, and selective reporting bias [26,32,35]. Of these, two studies were classified as having a high risk of bias, mainly due to their insufficient adjustment for crossover design elements and inadequate washout periods between interventions (Table 2) (Figure 3) [32,35].

Despite variations in the study methodology, most of the included studies contributed meaningful data regarding recovery time, patient satisfaction, and safety outcomes. However, studies that were identified as having a high risk of bias may have introduced some variability, particularly in the reported recovery times, contributing to the overall heterogeneity.

### 3.4. Meta-Analysis of Recovery Time

**Figure 4:**
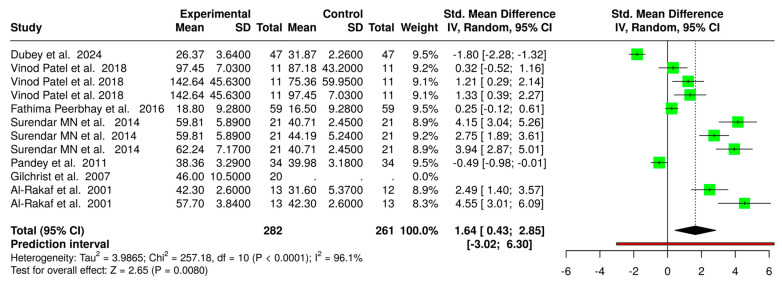
Forest plot of standardized mean differences (SMDs), with 95% confidence intervals (CIs), comparing outcomes between intervention and control groups across included studies in this systematic review. Each study is represented by a green square (point estimate), with the size of the square indicating the study’s relative weight in the meta-analysis. The horizontal lines denote 95% CIs. The vertical dashed line at an SMD of 0 represents the line of no effect. The pooled effect size is represented by the diamond at the bottom, showing a statistically significant overall effect in favor of the experimental group (SMD = 1.64; 95% CI: 0.43 to 2.85; Z = 2.65; and *p* = 0.0080). High heterogeneity was observed among the studies (I² = 96.1%; τ^2^ = 3.9865; and *p* < 0.0001), and the prediction interval (−3.02 to 6.30) indicated substantial variability in potential effects across different settings or populations [16,18,20,28,31,33,36].

A total of seven studies, involving 282 participants in the experimental group and 261 in the control group, were included in the meta-analysis. Using a random-effects model with the inverse variance method, the pooled standardized mean difference (SMD) was 1.64 (95% CI: 0.43 to 2.85; *p* < 0.05), indicating a significant reduction in recovery time for the intervention group. However, high heterogeneity (Higgins I^2^ = 96.1%, *p* < 0.01) was observed, suggesting substantial variations in the effect sizes across the studies. The potential sources of this heterogeneity include differences in the intervention dosages, administration methods, patient characteristics, and study designs. Despite this variability, the overall effect suggests a clinically meaningful reduction in recovery time. The forest plot (Figure 4) provides a visual representation of the standardized mean difference for each study, along with the overall effect size.

Regarding adverse effects, most were mild to moderate in nature, with no life-threatening events reported. The most frequently observed adverse effects included nasal irritation, which was noted in five studies, particularly with midazolam [17,19,20,25,27]. Drowsiness and prolonged sedation were commonly seen in studies involving dexmedetomidine (seven studies) [20,21,23,25,26,31,33]. Mild nausea and vomiting were reported in three studies, particularly in the ketamine group [21,31,33]. Additionally, bradycardia was transiently observed in two studies using dexmedetomidine [19,24]. Overall, these findings suggest that intranasal sedation is generally safe, with adverse effects being transient and self-limiting.

### 3.5. Patient Satisfaction and Acceptance

Patient satisfaction was assessed qualitatively in fifteen studies, primarily through parental feedback and behavioral scales [16,17,18,19,20,21,22,23,26,27,31,32,33,34,35]. Several trends emerged from these assessments. Dexmedetomidine was associated with higher parental satisfaction, which was largely due to its ability to provide deeper sedation and a smoother recovery process [21,23,25]. Midazolam, while appreciated for its rapid onset, showed some variability in patient acceptance across the studies [20,27]. Ketamine was generally well accepted, with many parents noting its anxiolytic effects as particularly beneficial [19,24]. Overall, intranasal sedation techniques were well-tolerated and accepted by both children and caregivers, with high levels of acceptance reported across the studies.

### 3.6. Summary of Findings

Intranasal sedation effectively alleviates dental anxiety in pediatric patients. Midazolam and ketamine were associated with faster recovery times compared to dexmedetomidine. Adverse effects were generally mild and transient, with no severe complications reported. Parental satisfaction was high across all sedation methods, with dexmedetomidine offering smoother sedation; however, it was also linked to longer recovery times.

## 4. Discussion

This systematic review and meta-analysis was, to the best of our knowledge, the first to comprehensively evaluate and compare the efficacy, safety, and recovery outcomes of intranasally administered midazolam, dexmedetomidine, and ketamine for pediatric dental sedation. It has provided an evidence-based guide for clinical decision-making. Given the increasing demand for minimally invasive and child-friendly sedation methods in pediatric dentistry, this comparison offers practical insights for clinicians seeking tailored pharmacological strategies.

The meta-analysis of recovery time revealed that dexmedetomidine was associated with significantly longer recovery durations compared to both midazolam and ketamine, which was consistent with the findings of previous research studies [9,37]. Specifically, midazolam was linked to a faster recovery profile than dexmedetomidine, with a mean difference of −19.1 min (*p* < 0.05), supporting earlier studies that highlighted its rapid onset and shorter sedative effects [38]. Additionally, ketamine showed a quick recovery time compared to dexmedetomidine (mean difference: −15.6 min, *p* < 0.05), aligning with the findings of Preethy et al. (2021), which emphasized ketamine’s dissociative sedation and rapid clearance [10]. These differences in recovery profiles are particularly relevant in outpatient settings with frequent patient flow, where timely discharge and efficient scheduling are important for maintaining clinical efficiency.

Patient satisfaction, which was primarily assessed through parental feedback, was highest with dexmedetomidine, which provided smoother sedation and reduced emergence agitation. This is consistent with previous studies, which reported superior parental satisfaction with dexmedetomidine, despite its association with prolonged sedation [39,40]. In contrast, midazolam was favored in situations requiring a faster onset of sedation. However, it demonstrated higher variability in patient acceptance, which was partly due to occasional paradoxical reactions [41,42]. Such reactions, though relatively uncommon, can complicate behavior management and may necessitate closer monitoring or alternative sedation planning. Ketamine was generally well-accepted, with many parents appreciating its anxiolytic effects. However, some studies noted concerns related to hypersalivation and mild postoperative agitation [42]. These findings underscore the need for individualized sedation strategies, taking into account both patient-specific factors and the procedural requirements [5,41].

The qualitative synthesis of adverse effects revealed that complications were generally mild-to-moderate, with no life-threatening events reported. The most frequently observed adverse effects included nasal irritation, which caused brief discomfort; prolonged sedation, which required extended monitoring; and transient bradycardia, which did not lead to significant hemodynamic instability. Notably, the occurrence of bradycardia was commonly associated with dexmedetomidine, highlighting the importance of careful cardiovascular monitoring during and after its use to ensure patient safety. These findings are consistent with the American Academy of Pediatric Dentistry’s (AAPD’s) sedation guidelines, which emphasize the importance of post-procedural monitoring, particularly in cases involving the use of dexmedetomidine [43].

Previous reviews have largely focused on overall sedation success, often overlooking important post-sedation outcomes, such as recovery time and patient satisfaction. For instance, Swaminathan et al. (2025) confirmed the effectiveness of intranasal sedation in managing pediatric dental behavior but did not assess recovery time or satisfaction levels [9]. In contrast, Preethy et al. (2021) reported substantial variability in recovery times across different intranasal agents, highlighting the need for more standardized protocols in pediatric sedation practice [10].

### 4.1. Clinical Implications

These findings offer valuable clinical guidance for dental practitioners treating pediatric patients, aiding in the selection of the most appropriate intranasal sedative, based on procedural requirements and patient-specific considerations. Midazolam appears to be the most suitable option for shorter procedures, given its rapid onset and faster recovery profile; however, the potential for paradoxical reactions and nasal irritation should be taken into account. Dexmedetomidine, on the other hand, provides smoother sedation and higher levels of patient and parental satisfaction, but is associated with prolonged recovery times, making it more appropriate for longer or more complex procedures. Ketamine offers a balanced profile of sedation and analgesia, with a faster recovery than dexmedetomidine, although, clinicians should anticipate side effects such as hypersalivation. These findings align with the American Academy of Pediatric Dentistry’s (AAPD’s) guidelines, which emphasize individualized sedation planning based on procedure duration, patient cooperation, and overall safety [43].

### 4.2. Limitations and Future Directions

This study presented some limitations that should be acknowledged. Considerable heterogeneity across the included studies, particularly in dosing regimens and outcome assessment tools, may have influenced the overall findings. Additionally, the absence of standard deviation data in three studies necessitated estimations based on comparable trials, which may have introduced a degree of bias. Another notable limitation was the lack of data on long-term behavioral outcomes, underscoring the need for longitudinal research in this area. Crossover trials also presented specific challenges in bias assessment, as potential carryover effects and variations between study periods may have affected the interpretation of intranasal sedation recovery time outcomes.

The overall quality of the evidence across the included studies varied. While three studies were assessed as having a low risk of bias, twelve raised some concerns, and six were classified as having a high risk of bias. The most frequently encountered issues included the inconsistent reporting of patient satisfaction outcomes and variations in sedation scoring systems, which may have introduced subjectivity into the qualitative evaluations. Despite these concerns, studies were not excluded solely based on their risk-of- bias ratings. This decision aligned with the established systematic review guidelines, which caution against excluding studies solely due to quality concerns, as this can introduce selection bias. Instead, we aimed to present a comprehensive synthesis of the available evidence, while transparently acknowledging methodological limitations. Including all eligible studies allowed for a more balanced and inclusive interpretation of the current literature. To account for potential bias, we carefully considered the risk of bias in our analysis and interpretation of the findings, and we emphasized the need for future research to address these methodological shortcomings.

Despite these limitations, the majority of the included studies adhered to well-structured randomized controlled trial designs, contributing meaningful evidence to the field. Future research should prioritize greater standardization of sedation protocols, consistent and transparent outcome reporting, and improved methodological rigor in crossover trial designs. Furthermore, efforts should be made to develop evidence-based dosing guidelines for intranasal sedation in pediatric dentistry, exploring the long-term behavioral and cognitive effects of sedation. Additionally, comparative studies evaluating intranasal, oral, and intravenous sedation routes are essential to determine the most effective and patient-appropriate approaches for various clinical scenarios in pediatric dentistry.

## 5. Conclusions

This systematic review and meta-analysis has offered valuable insights into the effectiveness of intranasal sedation in pediatric dentistry, specifically in terms of recovery time, patient satisfaction, and safety profiles. The findings indicated that midazolam is associated with a more rapid recovery, making it suitable for shorter procedures, while dexmedetomidine delivers smoother and more stable sedation, but necessitates extended post-procedural monitoring. Ketamine presents a balanced profile, providing both sedation and analgesia with a moderate recovery timeline. Overall, intranasal sedation appears to be a safe and well-tolerated option for managing pediatric dental anxiety. However, the observed variations in recovery duration, patient acceptance, and minor adverse events highlight the need for personalized sedation strategies, which are tailored to the individual child and procedure. Future studies should focus on the development of standardized intranasal sedation protocols and the evaluation of long-term behavioral and cognitive outcomes to further enhance the quality and safety of pediatric dental care.

## Figures and Tables

**Table 1:** The revised Cochrane risk of bias tool (RoB 2) for parallel-group RCTs.

Study	Randomization Bias	Bias in Deviations from Intended Interventions	Bias Due to Missing Outcome Data	Bias in Measurement of Outcomes	Bias in Selection of Reported Results	Overall Bias Judgment
Elkhatib et al. (2024) [19]	Low	Low	Some concerns	Low	Low	Some concerns
Dubey et al. (2024) [20]	Low	Low	Low	Low	Some concerns	Some concerns
El-Rouby et al. (2024) [21]	Low	Low	Low	Low	Low	Low
Balaji S et al. (2024) [22]	Low	Low	Low	Low	Some concerns	Some concerns
Nie J et al. (2023) [23]	Low	Low	Some concerns	Low	Low	Some concerns
Elkhatib AA et al. (2022) [24]	Low	Some concerns	Low	Low	Low	Some concerns
Salem K et al. (2022) [25]	Low	Low	Low	Some concerns	Low	Some concerns
Khalil W, Raslan N (2020) [27]	Low	Low	Some concerns	Low	Low	Some concerns
Patel V (2018) [28]	Low	Low	Some concerns	Low	Low	Some concerns
Shanmugaavel AK. (2016) [29]	Low	Some concerns	Low	Some concerns	Low	High risk
Peerbhay F et al. (2016) [16]	Some concerns	Low	Low	Low	Low	Some concerns
Shanmugaavel AK. et al. (2015) [30]	Low	Low	Low	Low	Low	Low
Fallahinejad Ghajari M et al. (2015) [17]	Low	Some concerns	Low	Some concerns	Some concerns	High risk
Surendar MN et al. (2014) [31]	Low	Low	Some concerns	Low	Some concerns	High risk
Pandey et al. (2011) [33]	Low	Some concerns	Low	Low	Low	Some concerns
Mazaheri R et al. (2007) [34]	Some concerns	Low	Low	Low	Low	Some concerns
Gilchrist et al. (2007) [18]	Some concerns	Some concerns	Low	Some concerns	Low	High risk
Al-Rakaf et al. (2001) [36]	Low	Low	Low	Some concerns	Low	Some concerns

**Table 2:** The revised Cochrane risk of bias tool (RoB 2) for crossover RCTs.

Study	Randomization Bias	Carryover Effects	Period Effects	Bias Due to Missing Outcome Data	Bias in Measurement of Outcomes	Bias in Selection of Reported Results	Overall Bias Judgment
Shaat MA et al. (2021) [26]	Low	Low	Low	Low	Low	Low	Low
Sunbul N et al. (2014) [32]	Some concerns	Low	Low	Some concerns	Low	Some concerns	High risk
Dallman JA et al. (2001) [35]	Some concerns	Low	Low	Low	Low	Some concerns	High risk

## Data Availability

The original contributions presented in this study are included in the article. Additional data are available from the authors upon reasonable request.

## Supplementary Materials

The following supporting information can be downloaded at: https://www.mdpi.com/article/10.3390/jcm14124038/s1, Table S1: Characteristics of the included studies.
