# Supplementary material for: Recovery Time, Patient Satisfaction, and Safety of Intranasal Sedatives in Pediatric Dentistry: A Systematic Review and Meta-Analysis
Source: J Clin Med. 2025 Jun 7;14(12):4038. doi: 10.3390/jcm14124038 (PMC12194465; doi:10.3390/jcm14124038)
Supplement: Supplementary file 1 [file jcm-14-04038-s001.zip › jcm-3616350-SI.pdf]

S.no	Author, Year, Country	Study Design	Sample Size	Age Group	Intervention	Control Group	Outcomes Measured	Primary Findings	Adverse Effects
1.	El Khatib et al. (2024) Egypt [19]	RCT	n=72	4–6 years	Dexmedetomidine (5 µg/kg) vs. Dex + Midazolam (3 µg/kg + 0.3 mg/kg) vs. Midazolam (0.5 mg/kg)	Different intranasal sedation doses	Safety, Recovery Time, Patient Satisfaction	Intranasal sedation provided effective sedation with minimal adverse effects. Recovery ~35 min	Minimal, transient bradycardia in some patients
2.	Dubey et al. (2024), India [20]	RCT	n=47	3–9 years	Intranasal Ketamine (7 mg/kg) vs. Midazolam (0.3 mg/kg) + Dexmedetomidine (3 µg/kg)	Different sedation combinations	Safety, Recovery Time, Patient Satisfaction	Intranasal Ketamine had a shorter recovery (~26 min), while Midazolam + Dex prolonged sedation (~32 min)	No severe effects; mild nasal irritation noted
3.	El-Rouby et al. (2024), Egypt [21]	RCT	n=56	3–5 years	Buccal Dexmedetomidine (4 µg/kg) vs. Dex + Ketamine (2 µg/kg + 2 mg/kg)	Dexmedetomidine vs. Dex + Ketamine	Safety, Recovery Time, Patient Satisfaction	Dexmedetomidine + Ketamine resulted in longer sedation duration (~55 min) compared to Dex alone (~40 min)	Mild drowsiness, slight increase in salivation
4.	Balaji S et al. (2024), India [22]	RCT	n=80	4–10 years	Intranasal Midazolam (0.5 mg/kg)	No control (single-arm study)	Patient Satisfaction	Parent-administered intranasal sedation had slightly better acceptance than dentist-administered	No severe effects reported
5.	Nie J et al. (2023), China [23]	RCT	n=83	3–12 years	Intranasal Dexmedetomidine (2 µg/kg) + Oral Midazolam (0.5 mg/kg) vs. Oral Midazolam & intranasal placebo	Oral Midazolam vs. Oral Midazolam + Intranasal Dexmedetomidine	Safety, Recovery Time, Patient Satisfaction	Intranasal Dex + Oral Mid had slightly longer sedation time (~17.5 min) than Oral Mid alone (~15.7 min)	Lethargy was observed in Intranasal Dex + Oral Midazolam
6.	ElKhatib AA et al. (2022), Egypt [24]	Triple-Blind Randomized Controlled Trial (T-RCT)	n=72 (3 Groups: Dexmedetomidine, Dex + Midazolam, Midazolam)	4–6 years	Nebulized Dexmedetomidine (5 µg/kg) vs. Nebulized Dex (3 µg/kg) + Midazolam (0.3 mg/kg) vs. Nebulized Midazolam (0.5 mg/kg)	Different sedation regimens (Nebulized Dexmedetomidine vs. Midazolam vs. Combination)	Sedation Level, Analgesic Effect, Ease of Treatment Completion, Safety (Vital Signs)	Dexmedetomidine provided moderate sedation and better patient cooperation compared to Midazolam. Combination therapy resulted in deeper sedation but did not improve anxiolysis.	Mild bradycardia in Dexmedetomidine group; no significant respiratory depression in any group
7.	Salem K et al. (2022), Iran [25]	RCT	n=92	4- 6 years	Intranasal Midazolam (0.2 mg/kg) vs. Intranasal Dexmedetomidine (1 µg/kg)	Comparison between two intranasal sedatives	Sedation success, safety, patient behaviour	Midazolam resulted in more acceptable behaviour than dexmedetomidine (64% vs. 47.7%, p = 0.007); No major adverse effects	No significant adverse effects; Dexmedetomidine caused less nasal irritation
8.	Shaat MA et al. (2021), Egypt [26]	RCT	n=42 (Crossover design)	5-7 years	Intranasal Dexmedetomidine (1 µg/kg)	Sublingual Dexmedetomidine (1 µg/kg)	Sedation onset time, anxiety, patient acceptance,	Intranasal dexmedetomidine had faster onset time (9.87± 1.27 min) compared to	No significant adverse effects recorded

							post-operative response	sublingual (13.89± 1.54 min); No adverse effects	
9.	Khalil W, Raslan N. (2020), Iran [27]	Randomized Placebo-Controlled Trial	n=63 (3 Groups: Midazolam, Midazolam + Lidocaine, Placebo)	4–11 years	Intranasal Midazolam (0.5 mg/kg) with or without topical Lidocaine (2%)	Placebo (Saline) + Intranasal Midazolam (0.5 mg/kg)	Safety (Pain Scale), Patient Satisfaction (Behavioural Scales, Parental Acceptance)	Lidocaine spray significantly reduced nasal burning sensation and improved drug acceptance. Parental acceptance of intranasal sedation was highest in the Lidocaine group	No severe adverse effects; minor nasal irritation in the non-lidocaine group
10.	Patel V (2018), India [28]	RCT	n=44	4–9 years	Intranasal Dexmedetomidine (1–2 &2-2.5 μg/kg) vs. Oral Dexmedetomidine (3-4 & 4–5 μg/kg)	Oral vs. Intranasal Dexmedetomidine	Safety, Recovery Time, Patient behaviour	Oral Dexmedetomidine had significantly shorter recovery time while intranasal dose had faster onset	No adverse effects
11.	Shanmugaavel AK. (2016), India [29]	RCT	n=20	3-7 years	Intranasal Midazolam (0.2 mg/kg)	Sublingual Midazolam (0.2 mg/kg)	Dental anxiety, behaviour changes, salivary cortisol levels	Intranasal and sublingual midazolam were equally effective in reducing anxiety; Intranasal had a slightly deeper sedation effect	No significant adverse effects recorded
12.	Peerbhay F and Elsheikhomer AM. (2016), South Africa [16]	T-RCT	n=118 (Two Groups: 0.3 mg/kg INM, 0.5 mg/kg INM)	4–6 years	Intranasal Midazolam (0.3 mg/kg and 0.5 mg/kg) via mucosal atomizer device (MAD)	Comparison of different INM doses (0.3 mg/kg vs. 0.5 mg/kg)	Safety (Vital Signs), Recovery Time, Anxiety & Sedation Level (Wilson & Venham Scale), Patient Satisfaction	Both doses resulted in effective sedation. The 0.5 mg/kg dose showed better anxiety reduction but slightly prolonged recovery time (18.8 min vs. 16.5 min)	Mild burning sensation in 9% of children; transient oxygen desaturation in 1 case
13.	Shanmugaavel AK. et al. (2015), India [30]	RCT	n=40	3-7 years	Intranasal Midazolam (0.2 mg/kg)	Sublingual Midazolam (0.2 mg/kg)	Sedation onset time, patient behaviour, sedation efficacy	Intranasal midazolam had a significantly faster onset of action (9.40 ± 1.84 min) compared to sublingual (13.80 ± 2.04 min); No adverse effects	No significant adverse effects recorded
14.	Fallahinejad Ghajari M et al. (2015), Iran [17]	RCT	n=23	3-6 years	Intranasal Midazolam (0.5 mg/kg) + Ketamine (10 mg/kg)	Oral Midazolam (0.5 mg/kg) + Ketamine (10 mg/kg)	Sedation success, safety, recovery time, patient behaviour	Intranasal sedation resulted in better sedation efficacy and reduced movement; Recovery time was longer in the intranasal group	Nausea, vomiting, drowsiness, and reduced activity observed; No major complications
15.	Surendar MN et al. (2014), India [31]	RCT	n=84	4–14 years	Dexmedetomidine (1–1.5 μg/kg) vs. Midazolam (0.2 mg/kg) vs. Ketamine (5 mg/kg)	Different sedation drugs	Efficacy, Safety, Recovery Time,	Midazolam had faster onset but shorter sedation	Occasional vomiting with Ketamine and

							Patient Satisfaction	duration; Dex had longer recovery time (~60 min)	Dexmedetomidine group
16.	Sunbul N et al. (2014), Saudi Arabia [32]	RCT	n=25 (Crossover design)	3-6 years	Intranasal Midazolam (0.3 mg/kg)	Buccal Midazolam (0.3 mg/kg)	Sedation success, safety, recovery time, patient behaviour	Intranasal midazolam had faster onset compared to buccal; Less resistance to administration	No major adverse effects; Mild discomfort noted
17.	Pandey et al. (2011), India [33]	RCT	n=34	2–6 years	Intranasal Ketamine (6 mg/kg) as Drops vs. Atomized Spray	Different administration techniques (Drops vs. Spray)	Safety, Recovery Time, Patient behaviour	Atomized spray had slightly faster onset and shorter recovery (~38 min) compared to Drops (~40 min)	Vomiting was observed in drops and spray administration
18.	Mazaheri R et al. (2007), Iran [34]	RCT	n=30	3–5 years	Intranasal Midazolam (0.5 mg/kg)	No control (single-arm study)	Safety, Recovery Time, Patient Satisfaction	Intranasal Midazolam was effective, with a recovery time of ~50–60 min	No adverse effects were seen
19.	Gilchrist et al. (2007), United Kingdom [18]	RCT	n= 20	2- 9 years	Intranasal Midazolam (0.25 mg/kg)	None	Safety, Recovery Time, Patient Satisfaction	Midazolam provided adequate anxiolysis; Mean time to treatment start: 13 min, Mean discharge time: 46 min	Sneezing, coughing and vomiting were noted
20.	Dallman JA et al. (2001),United States of America [35]	RCT (Double-blind, Crossover)	n=31	26–58 months	Intranasal Midazolam (0.2 mg/kg) via atomizer	Oral Chloral Hydrate (62.5 mg/kg) + Promethazine (12.5 mg)	Safety, Recovery Time, Patient Satisfaction	Intranasal Midazolam was as safe and effective as Oral Chloral Hydrate + Promethazine and also had faster recovery	One case of vomiting in Chloral Hydrate group; mild nasal irritation and crying reported in Midazolam group
21.	Al-Rakaf et al. (2001), Saudi Arabia [36]	(RCT)	n=38	2-5 years	Intranasal Midazolam	Group A-0.3mg/Kg; Group B-0.4mg/Kg; Group C-0.5mg/Kg	Safety, Recovery Time, Patient Satisfaction	Midazolam was effective for conscious sedation	No adverse effect

Table S1. Comprehensive overview of the studies' characteristics

(Supplementary File S1)
